# Unspoken challenges: The hidden struggles of patients under surgical neuro-oncology care

**DOI:** 10.12669/pjms.41.13(PINS-NNOS).13503

**Published:** 2025-12

**Authors:** Muhammad Sohaib Shahid, Muhammad Fawad ul Hassan, Asif Bashir

**Affiliations:** 1 Muhammad Sohaib Shahid, Medical Student. Aga Khan University, Karachi, Pakistan; 2 Muhammad Fawad ul Hassan, MBBS. Department of Neurosurgery, Punjab Institute of Neurosciences, Lahore, Pakistan; 3 Asif Bashir, MD, FAANS, FACS, (Diplomate American Board of Neurosurgery). Department of Neurosurgery, Punjab Institute of Neurosciences, Lahore, Pakistan

**Keywords:** Brain neoplasm, Counseling, Neurosurgery

Central nervous system (CNS) tumors represent a significant global health burden, with their incidence affected by genetic and environmental factors alongside substantial underreporting in low and middle-income countries (LMICs).^1^ Among primary CNS tumors, meningiomas are the most common benign neoplasms (41.7%), and glioblastomas the most frequent malignant type (13.9%).^2^ Secondary CNS metastases constitute the largest share of cases, with an incidence of 24.2 per 100,000 population.^3^ Globally, a higher incidence has been observed in males.^4^ Significant regional discordance exists in brain tumor incidence. In Punjab, Pakistan, a crude estimated annual incidence rate of brain tumors is 0.21 per 100,000 population.^5^ In contrast, the global age-standardized incidence rate for primary malignant brain and other CNS tumors is reported at 3.5 per 100,000 population, likely due to robust surveillance systems and higher diagnostic sensitivity.^4^

Surgical intervention remains the cornerstone of treatment for CNS tumors, with various techniques. These include open, minimally invasive, endoscopic, and microscopic approaches, employed based on tumor type, size and location. Regardless of the surgical approach, patients remain at risk of complications. While some perioperative complications such as sensory and motor deficits, hydrocephalus, and seizures are well-documented in the literature, certain other sequelae remain largely unrecognized and underreported.

We wish to bring attention to those overlooked perioperative sequelae which are often considered trivial and thus dismissed in clinical discussions. Several aspects of the perioperative experience remain largely unaddressed despite their impact on patient well-being. Preoperative head shaving without prior discussion causes body image problems for patients unprepared for the change.^6^ Catheterization, though routine, can lead to catheter-related bladder discomfort.^7^ Intraoperatively, blood transfusions are often addressed only in terms of necessity, with limited attention to potential complications or patient preferences. Postoperative recovery presents further challenges. Heavy dressings add discomfort, and multiple intravenous and arterial lines may cause pain. Food and water restrictions further increase patient distress. The intensive care unit (ICU) setting itself can be overwhelming, with continuous noise, bright lights, and frequent interventions causing disruption in rest.

An open and transparent discussion of risks, benefits, and alternative treatments is fundamental to patient-centered care. The focus is currently primarily being placed on life-threatening or functionally debilitating complications. The omission of conversation regarding the aforementioned sequelae is often a result of these issues being perceived as minor. However, patients cannot make fully informed decisions about their care if they are unaware of these other factors. Neglecting to disclose such pertinent details raises ethical concerns, potentially conflicting with the medical ethical principles of autonomy and informed consent. A comprehensive preoperative discussion that includes these aspects can significantly enhance patient satisfaction, trust in the medical team, and overall perception of care. This is particularly crucial in neuro-oncology patients, who often face heightened emotional vulnerability due to the nature of their diagnosis and treatment.

Hence, we aim to highlight these perioperative sequelae in neuro-oncology patients. While often considered inconsequential, these factors continue to impact patient well-being. This letter underscores the need for a comprehensive preoperative discussion and transparent communication as key components of informed consent. Implementing this approach can enhance patient satisfaction and trust, ultimately improving the overall surgical experience for neuro-oncology patients.

Healthcare providers should be encouraged and trained to always disclose these seemingly insignificant sequelae that impact patients’ physical and psychological equilibrium. Comprehensive discussion of both major and minor complications should be performed. A detailed informed consent paper can be introduced as a part of the pre-operative discussion. The consent paper should hierarchically contain all the minor and major complications to ensure that the patients are fully informed about all aspects of care. Future studies should assess whether discussing these overlooked postoperative sequelae leads to measurable improvements in patient outcomes, satisfaction scores, psychological well-being, and physical comfort. Studying these outcomes could provide insights into the role of informed discussions in enhancing holistic patient care. Such findings might enhance patient care strategies and their overall well-being.

## Author`s Contribution:

**MSS:** Conception & design, collected and analyzed the data, interpreted the results and drafted the manuscript.

**MFH:** Analyzed the data, interpreted the results and drafted the manuscript.

**AB:** Data analysis, critically reviewed and supervised the study.

All authors have approved the final version to be published and agreement to be accountable for all aspects of the work in ensuring that questions related to the accuracy or integrity of any part of the work are appropriately investigated and resolved.

## References

[1] Mbi Feh MK, Lyon KA, Brahmaroutu AV, Tadipatri R, Fonkem E (2021). The need for a central brain tumor registry in Africa:a review of central nervous system tumors in Africa from 1960 to 2017. Neurooncol Pract.

[2] Price M, Ballard C, Benedetti J, Neff C, Cioffi G, Waite KA (2024). CBTRUS statistical report:primary brain and other central nervous system tumors diagnosed in the United States in 2017–2021. Neuro Oncol.

[3] Habbous S, Forster K, Darling G, Jerzak K, Holloway CMB, Sahgal A (2020). Incidence and real-world burden of brain metastases from solid tumors and hematologic malignancies in Ontario:a population-based study. Neurooncol Adv.

[4] CBTRUS Fact Sheet 2024 - CBTRUS [Internet] (2024). CBTRUS.

[5] Masood K, Masood A, Zafar J, Shahid A, Kamran M, Murad S (2015). Trends and analysis of cancer incidence for common male and female cancers in the population of Punjab province of Pakistan during 1984 to 2014. Asian Pac J Cancer Prev.

[6] Yang SH, Cheng HC (2014). The influence of hair shaving on surgical site infections and body image in craniotomy. Int J Evid Based Healthc.

[7] Bai Y, Wang X, Li X, Pu C, Yuan H, Tang Y (2015). Management of catheter-related bladder discomfort in patients who underwent elective surgery. J Endourol.

